# Supplementary material for: OpenMM 7: Rapid development of high performance algorithms for molecular dynamics
Source: PLoS Comput Biol. 2017 Jul 26;13(7):e1005659. doi: 10.1371/journal.pcbi.1005659 (PMC5549999; doi:10.1371/journal.pcbi.1005659)
Supplement: S1 Source Code — (ZIP) [file pcbi.1005659.s002.zip › OpenMM-7.0.1-Source/docs/api-c++/genindex.html]

Index — OpenMM @OPENMM\_MAJOR\_VERSION@.@OPENMM\_MINOR\_VERSION@ documentation


# Index

**O**

## O

|  |  |
| --- | --- |
| OpenMM::AmoebaAngleForce (C++ class)  OpenMM::AmoebaAngleForce::addAngle (C++ function)  OpenMM::AmoebaAngleForce::AmoebaAngleForce (C++ function)  OpenMM::AmoebaAngleForce::getAmoebaGlobalAngleCubic (C++ function)  OpenMM::AmoebaAngleForce::getAmoebaGlobalAnglePentic (C++ function)  OpenMM::AmoebaAngleForce::getAmoebaGlobalAngleQuartic (C++ function)  OpenMM::AmoebaAngleForce::getAmoebaGlobalAngleSextic (C++ function)  OpenMM::AmoebaAngleForce::getAngleParameters (C++ function)  OpenMM::AmoebaAngleForce::getNumAngles (C++ function)  OpenMM::AmoebaAngleForce::setAmoebaGlobalAngleCubic (C++ function)  OpenMM::AmoebaAngleForce::setAmoebaGlobalAnglePentic (C++ function)  OpenMM::AmoebaAngleForce::setAmoebaGlobalAngleQuartic (C++ function)  OpenMM::AmoebaAngleForce::setAmoebaGlobalAngleSextic (C++ function)  OpenMM::AmoebaAngleForce::setAngleParameters (C++ function)  OpenMM::AmoebaAngleForce::updateParametersInContext (C++ function)  OpenMM::AmoebaAngleForce::usesPeriodicBoundaryConditions (C++ function)  OpenMM::AmoebaBondForce (C++ class)  OpenMM::AmoebaBondForce::addBond (C++ function)  OpenMM::AmoebaBondForce::AmoebaBondForce (C++ function)  OpenMM::AmoebaBondForce::getAmoebaGlobalBondCubic (C++ function)  OpenMM::AmoebaBondForce::getAmoebaGlobalBondQuartic (C++ function)  OpenMM::AmoebaBondForce::getBondParameters (C++ function)  OpenMM::AmoebaBondForce::getNumBonds (C++ function)  OpenMM::AmoebaBondForce::setAmoebaGlobalBondCubic (C++ function)  OpenMM::AmoebaBondForce::setAmoebaGlobalBondQuartic (C++ function)  OpenMM::AmoebaBondForce::setBondParameters (C++ function)  OpenMM::AmoebaBondForce::updateParametersInContext (C++ function)  OpenMM::AmoebaBondForce::usesPeriodicBoundaryConditions (C++ function)  OpenMM::AmoebaGeneralizedKirkwoodForce (C++ class)  OpenMM::AmoebaGeneralizedKirkwoodForce::addParticle (C++ function)  OpenMM::AmoebaGeneralizedKirkwoodForce::AmoebaGeneralizedKirkwoodForce (C++ function)  OpenMM::AmoebaGeneralizedKirkwoodForce::getIncludeCavityTerm (C++ function)  OpenMM::AmoebaGeneralizedKirkwoodForce::getNumParticles (C++ function)  OpenMM::AmoebaGeneralizedKirkwoodForce::getParticleParameters (C++ function)  OpenMM::AmoebaGeneralizedKirkwoodForce::getProbeRadius (C++ function)  OpenMM::AmoebaGeneralizedKirkwoodForce::getSoluteDielectric (C++ function)  OpenMM::AmoebaGeneralizedKirkwoodForce::getSolventDielectric (C++ function)  OpenMM::AmoebaGeneralizedKirkwoodForce::getSurfaceAreaFactor (C++ function)  OpenMM::AmoebaGeneralizedKirkwoodForce::setIncludeCavityTerm (C++ function)  OpenMM::AmoebaGeneralizedKirkwoodForce::setParticleParameters (C++ function)  OpenMM::AmoebaGeneralizedKirkwoodForce::setProbeRadius (C++ function)  OpenMM::AmoebaGeneralizedKirkwoodForce::setSoluteDielectric (C++ function)  OpenMM::AmoebaGeneralizedKirkwoodForce::setSolventDielectric (C++ function)  OpenMM::AmoebaGeneralizedKirkwoodForce::setSurfaceAreaFactor (C++ function)  OpenMM::AmoebaGeneralizedKirkwoodForce::updateParametersInContext (C++ function)  OpenMM::AmoebaGeneralizedKirkwoodForce::usesPeriodicBoundaryConditions (C++ function)  OpenMM::AmoebaInPlaneAngleForce (C++ class)  OpenMM::AmoebaInPlaneAngleForce::addAngle (C++ function)  OpenMM::AmoebaInPlaneAngleForce::AmoebaInPlaneAngleForce (C++ function)  OpenMM::AmoebaInPlaneAngleForce::getAmoebaGlobalInPlaneAngleCubic (C++ function)  OpenMM::AmoebaInPlaneAngleForce::getAmoebaGlobalInPlaneAnglePentic (C++ function)  OpenMM::AmoebaInPlaneAngleForce::getAmoebaGlobalInPlaneAngleQuartic (C++ function)  OpenMM::AmoebaInPlaneAngleForce::getAmoebaGlobalInPlaneAngleSextic (C++ function)  OpenMM::AmoebaInPlaneAngleForce::getAngleParameters (C++ function)  OpenMM::AmoebaInPlaneAngleForce::getNumAngles (C++ function)  OpenMM::AmoebaInPlaneAngleForce::setAmoebaGlobalInPlaneAngleCubic (C++ function)  OpenMM::AmoebaInPlaneAngleForce::setAmoebaGlobalInPlaneAnglePentic (C++ function)  OpenMM::AmoebaInPlaneAngleForce::setAmoebaGlobalInPlaneAngleQuartic (C++ function)  OpenMM::AmoebaInPlaneAngleForce::setAmoebaGlobalInPlaneAngleSextic (C++ function)  OpenMM::AmoebaInPlaneAngleForce::setAngleParameters (C++ function)  OpenMM::AmoebaInPlaneAngleForce::updateParametersInContext (C++ function)  OpenMM::AmoebaInPlaneAngleForce::usesPeriodicBoundaryConditions (C++ function)  OpenMM::AmoebaMultipoleForce (C++ class)  OpenMM::AmoebaMultipoleForce::addMultipole (C++ function)  OpenMM::AmoebaMultipoleForce::AmoebaMultipoleForce (C++ function)  OpenMM::AmoebaMultipoleForce::getAEwald (C++ function)  OpenMM::AmoebaMultipoleForce::getCovalentMap (C++ function)  OpenMM::AmoebaMultipoleForce::getCovalentMaps (C++ function)  OpenMM::AmoebaMultipoleForce::getCutoffDistance (C++ function)  OpenMM::AmoebaMultipoleForce::getElectrostaticPotential (C++ function)  OpenMM::AmoebaMultipoleForce::getEwaldErrorTolerance (C++ function)  OpenMM::AmoebaMultipoleForce::getExtrapolationCoefficients (C++ function)  OpenMM::AmoebaMultipoleForce::getInducedDipoles (C++ function)  OpenMM::AmoebaMultipoleForce::getMultipoleParameters (C++ function)  OpenMM::AmoebaMultipoleForce::getMutualInducedMaxIterations (C++ function)  OpenMM::AmoebaMultipoleForce::getMutualInducedTargetEpsilon (C++ function)  OpenMM::AmoebaMultipoleForce::getNonbondedMethod (C++ function)  OpenMM::AmoebaMultipoleForce::getNumMultipoles (C++ function)  OpenMM::AmoebaMultipoleForce::getPmeBSplineOrder (C++ function)  OpenMM::AmoebaMultipoleForce::getPmeGridDimensions (C++ function)  OpenMM::AmoebaMultipoleForce::getPMEParametersInContext (C++ function)  OpenMM::AmoebaMultipoleForce::getPolarizationType (C++ function)  OpenMM::AmoebaMultipoleForce::getSystemMultipoleMoments (C++ function)  OpenMM::AmoebaMultipoleForce::setAEwald (C++ function)  OpenMM::AmoebaMultipoleForce::setCovalentMap (C++ function)  OpenMM::AmoebaMultipoleForce::setCutoffDistance (C++ function)  OpenMM::AmoebaMultipoleForce::setEwaldErrorTolerance (C++ function)  OpenMM::AmoebaMultipoleForce::setExtrapolationCoefficients (C++ function)  OpenMM::AmoebaMultipoleForce::setMultipoleParameters (C++ function)  OpenMM::AmoebaMultipoleForce::setMutualInducedMaxIterations (C++ function)  OpenMM::AmoebaMultipoleForce::setMutualInducedTargetEpsilon (C++ function)  OpenMM::AmoebaMultipoleForce::setNonbondedMethod (C++ function)  OpenMM::AmoebaMultipoleForce::setPmeGridDimensions (C++ function)  OpenMM::AmoebaMultipoleForce::setPolarizationType (C++ function)  OpenMM::AmoebaMultipoleForce::updateParametersInContext (C++ function)  OpenMM::AmoebaMultipoleForce::usesPeriodicBoundaryConditions (C++ function)  OpenMM::AmoebaOutOfPlaneBendForce (C++ class)  OpenMM::AmoebaOutOfPlaneBendForce::addOutOfPlaneBend (C++ function)  OpenMM::AmoebaOutOfPlaneBendForce::AmoebaOutOfPlaneBendForce (C++ function)  OpenMM::AmoebaOutOfPlaneBendForce::getAmoebaGlobalOutOfPlaneBendCubic (C++ function)  OpenMM::AmoebaOutOfPlaneBendForce::getAmoebaGlobalOutOfPlaneBendPentic (C++ function)  OpenMM::AmoebaOutOfPlaneBendForce::getAmoebaGlobalOutOfPlaneBendQuartic (C++ function)  OpenMM::AmoebaOutOfPlaneBendForce::getAmoebaGlobalOutOfPlaneBendSextic (C++ function)  OpenMM::AmoebaOutOfPlaneBendForce::getNumOutOfPlaneBends (C++ function)  OpenMM::AmoebaOutOfPlaneBendForce::getOutOfPlaneBendParameters (C++ function)  OpenMM::AmoebaOutOfPlaneBendForce::setAmoebaGlobalOutOfPlaneBendCubic (C++ function)  OpenMM::AmoebaOutOfPlaneBendForce::setAmoebaGlobalOutOfPlaneBendPentic (C++ function)  OpenMM::AmoebaOutOfPlaneBendForce::setAmoebaGlobalOutOfPlaneBendQuartic (C++ function)  OpenMM::AmoebaOutOfPlaneBendForce::setAmoebaGlobalOutOfPlaneBendSextic (C++ function)  OpenMM::AmoebaOutOfPlaneBendForce::setOutOfPlaneBendParameters (C++ function)  OpenMM::AmoebaOutOfPlaneBendForce::updateParametersInContext (C++ function)  OpenMM::AmoebaOutOfPlaneBendForce::usesPeriodicBoundaryConditions (C++ function)  OpenMM::AmoebaPiTorsionForce (C++ class)  OpenMM::AmoebaPiTorsionForce::addPiTorsion (C++ function)  OpenMM::AmoebaPiTorsionForce::AmoebaPiTorsionForce (C++ function)  OpenMM::AmoebaPiTorsionForce::getNumPiTorsions (C++ function)  OpenMM::AmoebaPiTorsionForce::getPiTorsionParameters (C++ function)  OpenMM::AmoebaPiTorsionForce::setPiTorsionParameters (C++ function)  OpenMM::AmoebaPiTorsionForce::updateParametersInContext (C++ function)  OpenMM::AmoebaPiTorsionForce::usesPeriodicBoundaryConditions (C++ function)  OpenMM::AmoebaStretchBendForce (C++ class)  OpenMM::AmoebaStretchBendForce::addStretchBend (C++ function)  OpenMM::AmoebaStretchBendForce::AmoebaStretchBendForce (C++ function)  OpenMM::AmoebaStretchBendForce::getNumStretchBends (C++ function)  OpenMM::AmoebaStretchBendForce::getStretchBendParameters (C++ function)  OpenMM::AmoebaStretchBendForce::setStretchBendParameters (C++ function)  OpenMM::AmoebaStretchBendForce::updateParametersInContext (C++ function)  OpenMM::AmoebaStretchBendForce::usesPeriodicBoundaryConditions (C++ function)  OpenMM::AmoebaTorsionTorsionForce (C++ class)  OpenMM::AmoebaTorsionTorsionForce::addTorsionTorsion (C++ function)  OpenMM::AmoebaTorsionTorsionForce::AmoebaTorsionTorsionForce (C++ function)  OpenMM::AmoebaTorsionTorsionForce::getNumTorsionTorsionGrids (C++ function)  OpenMM::AmoebaTorsionTorsionForce::getNumTorsionTorsions (C++ function)  OpenMM::AmoebaTorsionTorsionForce::getTorsionTorsionGrid (C++ function)  OpenMM::AmoebaTorsionTorsionForce::getTorsionTorsionParameters (C++ function)  OpenMM::AmoebaTorsionTorsionForce::setTorsionTorsionGrid (C++ function)  OpenMM::AmoebaTorsionTorsionForce::setTorsionTorsionParameters (C++ function)  OpenMM::AmoebaTorsionTorsionForce::usesPeriodicBoundaryConditions (C++ function)  OpenMM::AmoebaVdwForce (C++ class)  OpenMM::AmoebaVdwForce::addParticle (C++ function)  OpenMM::AmoebaVdwForce::AmoebaVdwForce (C++ function)  OpenMM::AmoebaVdwForce::getCutoff (C++ function)  OpenMM::AmoebaVdwForce::getEpsilonCombiningRule (C++ function)  OpenMM::AmoebaVdwForce::getNonbondedMethod (C++ function)  OpenMM::AmoebaVdwForce::getNumParticles (C++ function)  OpenMM::AmoebaVdwForce::getParticleExclusions (C++ function)  OpenMM::AmoebaVdwForce::getParticleParameters (C++ function)  OpenMM::AmoebaVdwForce::getSigmaCombiningRule (C++ function)  OpenMM::AmoebaVdwForce::getUseDispersionCorrection (C++ function)  OpenMM::AmoebaVdwForce::setCutoff (C++ function)  OpenMM::AmoebaVdwForce::setEpsilonCombiningRule (C++ function)  OpenMM::AmoebaVdwForce::setNonbondedMethod (C++ function)  OpenMM::AmoebaVdwForce::setParticleExclusions (C++ function)  OpenMM::AmoebaVdwForce::setParticleParameters (C++ function)  OpenMM::AmoebaVdwForce::setSigmaCombiningRule (C++ function)  OpenMM::AmoebaVdwForce::setUseDispersionCorrection (C++ function)  OpenMM::AmoebaVdwForce::updateParametersInContext (C++ function)  OpenMM::AmoebaVdwForce::usesPeriodicBoundaryConditions (C++ function)  OpenMM::AmoebaWcaDispersionForce (C++ class)  OpenMM::AmoebaWcaDispersionForce::addParticle (C++ function)  OpenMM::AmoebaWcaDispersionForce::AmoebaWcaDispersionForce (C++ function)  OpenMM::AmoebaWcaDispersionForce::getAwater (C++ function)  OpenMM::AmoebaWcaDispersionForce::getDispoff (C++ function)  OpenMM::AmoebaWcaDispersionForce::getEpsh (C++ function)  OpenMM::AmoebaWcaDispersionForce::getEpso (C++ function)  OpenMM::AmoebaWcaDispersionForce::getNumParticles (C++ function)  OpenMM::AmoebaWcaDispersionForce::getParticleParameters (C++ function)  OpenMM::AmoebaWcaDispersionForce::getRminh (C++ function)  OpenMM::AmoebaWcaDispersionForce::getRmino (C++ function)  OpenMM::AmoebaWcaDispersionForce::getShctd (C++ function)  OpenMM::AmoebaWcaDispersionForce::getSlevy (C++ function)  OpenMM::AmoebaWcaDispersionForce::setAwater (C++ function)  OpenMM::AmoebaWcaDispersionForce::setDispoff (C++ function)  OpenMM::AmoebaWcaDispersionForce::setEpsh (C++ function)  OpenMM::AmoebaWcaDispersionForce::setEpso (C++ function)  OpenMM::AmoebaWcaDispersionForce::setParticleParameters (C++ function)  OpenMM::AmoebaWcaDispersionForce::setRminh (C++ function)  OpenMM::AmoebaWcaDispersionForce::setRmino (C++ function)  OpenMM::AmoebaWcaDispersionForce::setShctd (C++ function)  OpenMM::AmoebaWcaDispersionForce::setSlevy (C++ function)  OpenMM::AmoebaWcaDispersionForce::updateParametersInContext (C++ function)  OpenMM::AmoebaWcaDispersionForce::usesPeriodicBoundaryConditions (C++ function)  OpenMM::AndersenThermostat (C++ class)  OpenMM::AndersenThermostat::AndersenThermostat (C++ function)  OpenMM::AndersenThermostat::CollisionFrequency (C++ function)  OpenMM::AndersenThermostat::getDefaultCollisionFrequency (C++ function)  OpenMM::AndersenThermostat::getDefaultTemperature (C++ function)  OpenMM::AndersenThermostat::getRandomNumberSeed (C++ function)  OpenMM::AndersenThermostat::setDefaultCollisionFrequency (C++ function)  OpenMM::AndersenThermostat::setDefaultTemperature (C++ function)  OpenMM::AndersenThermostat::setRandomNumberSeed (C++ function)  OpenMM::AndersenThermostat::Temperature (C++ function)  OpenMM::AndersenThermostat::usesPeriodicBoundaryConditions (C++ function)  OpenMM::BrownianIntegrator (C++ class)  OpenMM::BrownianIntegrator::BrownianIntegrator (C++ function)  OpenMM::BrownianIntegrator::getFriction (C++ function)  OpenMM::BrownianIntegrator::getRandomNumberSeed (C++ function)  OpenMM::BrownianIntegrator::getTemperature (C++ function)  OpenMM::BrownianIntegrator::setFriction (C++ function)  OpenMM::BrownianIntegrator::setRandomNumberSeed (C++ function)  OpenMM::BrownianIntegrator::setTemperature (C++ function)  OpenMM::BrownianIntegrator::step (C++ function)  OpenMM::CMAPTorsionForce (C++ class)  OpenMM::CMAPTorsionForce::addMap (C++ function)  OpenMM::CMAPTorsionForce::addTorsion (C++ function)  OpenMM::CMAPTorsionForce::CMAPTorsionForce (C++ function)  OpenMM::CMAPTorsionForce::getMapParameters (C++ function)  OpenMM::CMAPTorsionForce::getNumMaps (C++ function)  OpenMM::CMAPTorsionForce::getNumTorsions (C++ function)  OpenMM::CMAPTorsionForce::getTorsionParameters (C++ function)  OpenMM::CMAPTorsionForce::setMapParameters (C++ function)  OpenMM::CMAPTorsionForce::setTorsionParameters (C++ function)  OpenMM::CMAPTorsionForce::updateParametersInContext (C++ function)  OpenMM::CMAPTorsionForce::usesPeriodicBoundaryConditions (C++ function)  OpenMM::CMMotionRemover (C++ class)  OpenMM::CMMotionRemover::CMMotionRemover (C++ function)  OpenMM::CMMotionRemover::getFrequency (C++ function)  OpenMM::CMMotionRemover::setFrequency (C++ function)  OpenMM::CMMotionRemover::usesPeriodicBoundaryConditions (C++ function)  OpenMM::CompoundIntegrator (C++ class)  OpenMM::CompoundIntegrator::addIntegrator (C++ function)  OpenMM::CompoundIntegrator::CompoundIntegrator (C++ function)  OpenMM::CompoundIntegrator::getConstraintTolerance (C++ function)  OpenMM::CompoundIntegrator::getCurrentIntegrator (C++ function)  OpenMM::CompoundIntegrator::getIntegrator (C++ function), [1]  OpenMM::CompoundIntegrator::getNumIntegrators (C++ function)  OpenMM::CompoundIntegrator::getStepSize (C++ function)  OpenMM::CompoundIntegrator::setConstraintTolerance (C++ function)  OpenMM::CompoundIntegrator::setCurrentIntegrator (C++ function)  OpenMM::CompoundIntegrator::setStepSize (C++ function)  OpenMM::CompoundIntegrator::step (C++ function)  OpenMM::CompoundIntegrator::~CompoundIntegrator (C++ function)  OpenMM::Context (C++ class)  OpenMM::Context::applyConstraints (C++ function)  OpenMM::Context::applyVelocityConstraints (C++ function)  OpenMM::Context::computeVirtualSites (C++ function)  OpenMM::Context::Context (C++ function), [1], [2]  OpenMM::Context::createCheckpoint (C++ function)  OpenMM::Context::getIntegrator (C++ function), [1]  OpenMM::Context::getMolecules (C++ function)  OpenMM::Context::getParameter (C++ function)  OpenMM::Context::getParameters (C++ function)  OpenMM::Context::getPlatform (C++ function), [1]  OpenMM::Context::getState (C++ function)  OpenMM::Context::getSystem (C++ function)  OpenMM::Context::loadCheckpoint (C++ function)  OpenMM::Context::reinitialize (C++ function)  OpenMM::Context::setParameter (C++ function)  OpenMM::Context::setPeriodicBoxVectors (C++ function)  OpenMM::Context::setPositions (C++ function)  OpenMM::Context::setState (C++ function)  OpenMM::Context::setTime (C++ function)  OpenMM::Context::setVelocities (C++ function)  OpenMM::Context::setVelocitiesToTemperature (C++ function)  OpenMM::Context::~Context (C++ function)  OpenMM::Continuous1DFunction (C++ class)  OpenMM::Continuous1DFunction::Continuous1DFunction (C++ function)  OpenMM::Continuous1DFunction::Copy (C++ function)  OpenMM::Continuous1DFunction::getFunctionParameters (C++ function)  OpenMM::Continuous1DFunction::setFunctionParameters (C++ function)  OpenMM::Continuous2DFunction (C++ class)  OpenMM::Continuous2DFunction::Continuous2DFunction (C++ function)  OpenMM::Continuous2DFunction::Copy (C++ function)  OpenMM::Continuous2DFunction::getFunctionParameters (C++ function)  OpenMM::Continuous2DFunction::setFunctionParameters (C++ function)  OpenMM::Continuous3DFunction (C++ class)  OpenMM::Continuous3DFunction::Continuous3DFunction (C++ function)  OpenMM::Continuous3DFunction::Copy (C++ function)  OpenMM::Continuous3DFunction::getFunctionParameters (C++ function)  OpenMM::Continuous3DFunction::setFunctionParameters (C++ function)  OpenMM::CustomAngleForce (C++ class)  OpenMM::CustomAngleForce::addAngle (C++ function)  OpenMM::CustomAngleForce::addGlobalParameter (C++ function)  OpenMM::CustomAngleForce::addPerAngleParameter (C++ function)  OpenMM::CustomAngleForce::CustomAngleForce (C++ function)  OpenMM::CustomAngleForce::getAngleParameters (C++ function)  OpenMM::CustomAngleForce::getEnergyFunction (C++ function)  OpenMM::CustomAngleForce::getGlobalParameterDefaultValue (C++ function)  OpenMM::CustomAngleForce::getGlobalParameterName (C++ function)  OpenMM::CustomAngleForce::getNumAngles (C++ function)  OpenMM::CustomAngleForce::getNumGlobalParameters (C++ function)  OpenMM::CustomAngleForce::getNumPerAngleParameters (C++ function)  OpenMM::CustomAngleForce::getPerAngleParameterName (C++ function)  OpenMM::CustomAngleForce::setAngleParameters (C++ function)  OpenMM::CustomAngleForce::setEnergyFunction (C++ function)  OpenMM::CustomAngleForce::setGlobalParameterDefaultValue (C++ function)  OpenMM::CustomAngleForce::setGlobalParameterName (C++ function)  OpenMM::CustomAngleForce::setPerAngleParameterName (C++ function)  OpenMM::CustomAngleForce::updateParametersInContext (C++ function)  OpenMM::CustomAngleForce::usesPeriodicBoundaryConditions (C++ function)  OpenMM::CustomBondForce (C++ class)  OpenMM::CustomBondForce::addBond (C++ function)  OpenMM::CustomBondForce::addGlobalParameter (C++ function)  OpenMM::CustomBondForce::addPerBondParameter (C++ function)  OpenMM::CustomBondForce::CustomBondForce (C++ function)  OpenMM::CustomBondForce::getBondParameters (C++ function)  OpenMM::CustomBondForce::getEnergyFunction (C++ function)  OpenMM::CustomBondForce::getGlobalParameterDefaultValue (C++ function)  OpenMM::CustomBondForce::getGlobalParameterName (C++ function)  OpenMM::CustomBondForce::getNumBonds (C++ function)  OpenMM::CustomBondForce::getNumGlobalParameters (C++ function)  OpenMM::CustomBondForce::getNumPerBondParameters (C++ function)  OpenMM::CustomBondForce::getPerBondParameterName (C++ function)  OpenMM::CustomBondForce::setBondParameters (C++ function)  OpenMM::CustomBondForce::setEnergyFunction (C++ function)  OpenMM::CustomBondForce::setGlobalParameterDefaultValue (C++ function)  OpenMM::CustomBondForce::setGlobalParameterName (C++ function)  OpenMM::CustomBondForce::setPerBondParameterName (C++ function)  OpenMM::CustomBondForce::updateParametersInContext (C++ function)  OpenMM::CustomBondForce::usesPeriodicBoundaryConditions (C++ function)  OpenMM::CustomCentroidBondForce (C++ class)  OpenMM::CustomCentroidBondForce::addBond (C++ function)  OpenMM::CustomCentroidBondForce::addGlobalParameter (C++ function)  OpenMM::CustomCentroidBondForce::addGroup (C++ function)  OpenMM::CustomCentroidBondForce::addPerBondParameter (C++ function)  OpenMM::CustomCentroidBondForce::addTabulatedFunction (C++ function)  OpenMM::CustomCentroidBondForce::CustomCentroidBondForce (C++ function)  OpenMM::CustomCentroidBondForce::getBondParameters (C++ function)  OpenMM::CustomCentroidBondForce::getEnergyFunction (C++ function)  OpenMM::CustomCentroidBondForce::getGlobalParameterDefaultValue (C++ function)  OpenMM::CustomCentroidBondForce::getGlobalParameterName (C++ function)  OpenMM::CustomCentroidBondForce::getGroupParameters (C++ function)  OpenMM::CustomCentroidBondForce::getNumBonds (C++ function)  OpenMM::CustomCentroidBondForce::getNumFunctions (C++ function)  OpenMM::CustomCentroidBondForce::getNumGlobalParameters (C++ function)  OpenMM::CustomCentroidBondForce::getNumGroups (C++ function)  OpenMM::CustomCentroidBondForce::getNumGroupsPerBond (C++ function)  OpenMM::CustomCentroidBondForce::getNumPerBondParameters (C++ function)  OpenMM::CustomCentroidBondForce::getNumTabulatedFunctions (C++ function)  OpenMM::CustomCentroidBondForce::getPerBondParameterName (C++ function)  OpenMM::CustomCentroidBondForce::getTabulatedFunction (C++ function), [1]  OpenMM::CustomCentroidBondForce::getTabulatedFunctionName (C++ function)  OpenMM::CustomCentroidBondForce::setBondParameters (C++ function)  OpenMM::CustomCentroidBondForce::setEnergyFunction (C++ function)  OpenMM::CustomCentroidBondForce::setGlobalParameterDefaultValue (C++ function)  OpenMM::CustomCentroidBondForce::setGlobalParameterName (C++ function)  OpenMM::CustomCentroidBondForce::setGroupParameters (C++ function)  OpenMM::CustomCentroidBondForce::setPerBondParameterName (C++ function)  OpenMM::CustomCentroidBondForce::updateParametersInContext (C++ function)  OpenMM::CustomCentroidBondForce::usesPeriodicBoundaryConditions (C++ function)  OpenMM::CustomCentroidBondForce::~CustomCentroidBondForce (C++ function)  OpenMM::CustomCompoundBondForce (C++ class)  OpenMM::CustomCompoundBondForce::addBond (C++ function)  OpenMM::CustomCompoundBondForce::addFunction (C++ function)  OpenMM::CustomCompoundBondForce::addGlobalParameter (C++ function)  OpenMM::CustomCompoundBondForce::addPerBondParameter (C++ function)  OpenMM::CustomCompoundBondForce::addTabulatedFunction (C++ function)  OpenMM::CustomCompoundBondForce::CustomCompoundBondForce (C++ function)  OpenMM::CustomCompoundBondForce::getBondParameters (C++ function)  OpenMM::CustomCompoundBondForce::getEnergyFunction (C++ function)  OpenMM::CustomCompoundBondForce::getFunctionParameters (C++ function)  OpenMM::CustomCompoundBondForce::getGlobalParameterDefaultValue (C++ function)  OpenMM::CustomCompoundBondForce::getGlobalParameterName (C++ function)  OpenMM::CustomCompoundBondForce::getNumBonds (C++ function)  OpenMM::CustomCompoundBondForce::getNumFunctions (C++ function)  OpenMM::CustomCompoundBondForce::getNumGlobalParameters (C++ function)  OpenMM::CustomCompoundBondForce::getNumParticlesPerBond (C++ function)  OpenMM::CustomCompoundBondForce::getNumPerBondParameters (C++ function)  OpenMM::CustomCompoundBondForce::getNumTabulatedFunctions (C++ function)  OpenMM::CustomCompoundBondForce::getPerBondParameterName (C++ function)  OpenMM::CustomCompoundBondForce::getTabulatedFunction (C++ function), [1]  OpenMM::CustomCompoundBondForce::getTabulatedFunctionName (C++ function)  OpenMM::CustomCompoundBondForce::setBondParameters (C++ function)  OpenMM::CustomCompoundBondForce::setEnergyFunction (C++ function)  OpenMM::CustomCompoundBondForce::setFunctionParameters (C++ function)  OpenMM::CustomCompoundBondForce::setGlobalParameterDefaultValue (C++ function)  OpenMM::CustomCompoundBondForce::setGlobalParameterName (C++ function)  OpenMM::CustomCompoundBondForce::setPerBondParameterName (C++ function)  OpenMM::CustomCompoundBondForce::updateParametersInContext (C++ function)  OpenMM::CustomCompoundBondForce::usesPeriodicBoundaryConditions (C++ function)  OpenMM::CustomCompoundBondForce::~CustomCompoundBondForce (C++ function)  OpenMM::CustomExternalForce (C++ class)  OpenMM::CustomExternalForce::addGlobalParameter (C++ function)  OpenMM::CustomExternalForce::addParticle (C++ function)  OpenMM::CustomExternalForce::addPerParticleParameter (C++ function)  OpenMM::CustomExternalForce::CustomExternalForce (C++ function)  OpenMM::CustomExternalForce::getEnergyFunction (C++ function)  OpenMM::CustomExternalForce::getGlobalParameterDefaultValue (C++ function)  OpenMM::CustomExternalForce::getGlobalParameterName (C++ function)  OpenMM::CustomExternalForce::getNumGlobalParameters (C++ function)  OpenMM::CustomExternalForce::getNumParticles (C++ function)  OpenMM::CustomExternalForce::getNumPerParticleParameters (C++ function)  OpenMM::CustomExternalForce::getParticleParameters (C++ function)  OpenMM::CustomExternalForce::getPerParticleParameterName (C++ function)  OpenMM::CustomExternalForce::setEnergyFunction (C++ function)  OpenMM::CustomExternalForce::setGlobalParameterDefaultValue (C++ function)  OpenMM::CustomExternalForce::setGlobalParameterName (C++ function)  OpenMM::CustomExternalForce::setParticleParameters (C++ function)  OpenMM::CustomExternalForce::setPerParticleParameterName (C++ function)  OpenMM::CustomExternalForce::updateParametersInContext (C++ function)  OpenMM::CustomExternalForce::usesPeriodicBoundaryConditions (C++ function)  OpenMM::CustomGBForce (C++ class)  OpenMM::CustomGBForce::addComputedValue (C++ function)  OpenMM::CustomGBForce::addEnergyTerm (C++ function)  OpenMM::CustomGBForce::addExclusion (C++ function)  OpenMM::CustomGBForce::addFunction (C++ function)  OpenMM::CustomGBForce::addGlobalParameter (C++ function)  OpenMM::CustomGBForce::addParticle (C++ function)  OpenMM::CustomGBForce::addPerParticleParameter (C++ function)  OpenMM::CustomGBForce::addTabulatedFunction (C++ function)  OpenMM::CustomGBForce::CustomGBForce (C++ function)  OpenMM::CustomGBForce::getComputedValueParameters (C++ function)  OpenMM::CustomGBForce::getCutoffDistance (C++ function)  OpenMM::CustomGBForce::getEnergyTermParameters (C++ function)  OpenMM::CustomGBForce::getExclusionParticles (C++ function)  OpenMM::CustomGBForce::getFunctionParameters (C++ function)  OpenMM::CustomGBForce::getGlobalParameterDefaultValue (C++ function)  OpenMM::CustomGBForce::getGlobalParameterName (C++ function)  OpenMM::CustomGBForce::getNonbondedMethod (C++ function)  OpenMM::CustomGBForce::getNumComputedValues (C++ function)  OpenMM::CustomGBForce::getNumEnergyTerms (C++ function)  OpenMM::CustomGBForce::getNumExclusions (C++ function)  OpenMM::CustomGBForce::getNumFunctions (C++ function)  OpenMM::CustomGBForce::getNumGlobalParameters (C++ function)  OpenMM::CustomGBForce::getNumParticles (C++ function)  OpenMM::CustomGBForce::getNumPerParticleParameters (C++ function)  OpenMM::CustomGBForce::getNumTabulatedFunctions (C++ function)  OpenMM::CustomGBForce::getParticleParameters (C++ function)  OpenMM::CustomGBForce::getPerParticleParameterName (C++ function)  OpenMM::CustomGBForce::getTabulatedFunction (C++ function), [1]  OpenMM::CustomGBForce::getTabulatedFunctionName (C++ function)  OpenMM::CustomGBForce::setComputedValueParameters (C++ function)  OpenMM::CustomGBForce::setCutoffDistance (C++ function)  OpenMM::CustomGBForce::setEnergyTermParameters (C++ function)  OpenMM::CustomGBForce::setExclusionParticles (C++ function)  OpenMM::CustomGBForce::setFunctionParameters (C++ function)  OpenMM::CustomGBForce::setGlobalParameterDefaultValue (C++ function)  OpenMM::CustomGBForce::setGlobalParameterName (C++ function)  OpenMM::CustomGBForce::setNonbondedMethod (C++ function)  OpenMM::CustomGBForce::setParticleParameters (C++ function)  OpenMM::CustomGBForce::setPerParticleParameterName (C++ function)  OpenMM::CustomGBForce::updateParametersInContext (C++ function)  OpenMM::CustomGBForce::usesPeriodicBoundaryConditions (C++ function)  OpenMM::CustomGBForce::~CustomGBForce (C++ function)  OpenMM::CustomHbondForce (C++ class)  OpenMM::CustomHbondForce::addAcceptor (C++ function)  OpenMM::CustomHbondForce::addDonor (C++ function)  OpenMM::CustomHbondForce::addExclusion (C++ function)  OpenMM::CustomHbondForce::addFunction (C++ function)  OpenMM::CustomHbondForce::addGlobalParameter (C++ function)  OpenMM::CustomHbondForce::addPerAcceptorParameter (C++ function)  OpenMM::CustomHbondForce::addPerDonorParameter (C++ function)  OpenMM::CustomHbondForce::addTabulatedFunction (C++ function)  OpenMM::CustomHbondForce::CustomHbondForce (C++ function)  OpenMM::CustomHbondForce::getAcceptorParameters (C++ function)  OpenMM::CustomHbondForce::getCutoffDistance (C++ function)  OpenMM::CustomHbondForce::getDonorParameters (C++ function)  OpenMM::CustomHbondForce::getEnergyFunction (C++ function)  OpenMM::CustomHbondForce::getExclusionParticles (C++ function)  OpenMM::CustomHbondForce::getFunctionParameters (C++ function)  OpenMM::CustomHbondForce::getGlobalParameterDefaultValue (C++ function)  OpenMM::CustomHbondForce::getGlobalParameterName (C++ function)  OpenMM::CustomHbondForce::getNonbondedMethod (C++ function)  OpenMM::CustomHbondForce::getNumAcceptors (C++ function)  OpenMM::CustomHbondForce::getNumDonors (C++ function)  OpenMM::CustomHbondForce::getNumExclusions (C++ function)  OpenMM::CustomHbondForce::getNumFunctions (C++ function)  OpenMM::CustomHbondForce::getNumGlobalParameters (C++ function)  OpenMM::CustomHbondForce::getNumPerAcceptorParameters (C++ function)  OpenMM::CustomHbondForce::getNumPerDonorParameters (C++ function)  OpenMM::CustomHbondForce::getNumTabulatedFunctions (C++ function)  OpenMM::CustomHbondForce::getPerAcceptorParameterName (C++ function)  OpenMM::CustomHbondForce::getPerDonorParameterName (C++ function)  OpenMM::CustomHbondForce::getTabulatedFunction (C++ function), [1]  OpenMM::CustomHbondForce::getTabulatedFunctionName (C++ function)  OpenMM::CustomHbondForce::setAcceptorParameters (C++ function)  OpenMM::CustomHbondForce::setCutoffDistance (C++ function)  OpenMM::CustomHbondForce::setDonorParameters (C++ function)  OpenMM::CustomHbondForce::setEnergyFunction (C++ function)  OpenMM::CustomHbondForce::setExclusionParticles (C++ function)  OpenMM::CustomHbondForce::setFunctionParameters (C++ function)  OpenMM::CustomHbondForce::setGlobalParameterDefaultValue (C++ function)  OpenMM::CustomHbondForce::setGlobalParameterName (C++ function)  OpenMM::CustomHbondForce::setNonbondedMethod (C++ function)  OpenMM::CustomHbondForce::setPerAcceptorParameterName (C++ function)  OpenMM::CustomHbondForce::setPerDonorParameterName (C++ function)  OpenMM::CustomHbondForce::updateParametersInContext (C++ function)  OpenMM::CustomHbondForce::usesPeriodicBoundaryConditions (C++ function)  OpenMM::CustomHbondForce::~CustomHbondForce (C++ function)  OpenMM::CustomIntegrator (C++ class)  OpenMM::CustomIntegrator::addComputeGlobal (C++ function)  OpenMM::CustomIntegrator::addComputePerDof (C++ function)  OpenMM::CustomIntegrator::addComputeSum (C++ function)  OpenMM::CustomIntegrator::addConstrainPositions (C++ function)  OpenMM::CustomIntegrator::addConstrainVelocities (C++ function)  OpenMM::CustomIntegrator::addGlobalVariable (C++ function)  OpenMM::CustomIntegrator::addPerDofVariable (C++ function)  OpenMM::CustomIntegrator::addUpdateContextState (C++ function)  OpenMM::CustomIntegrator::beginIfBlock (C++ function)  OpenMM::CustomIntegrator::beginWhileBlock (C++ function)  OpenMM::CustomIntegrator::CustomIntegrator (C++ function)  OpenMM::CustomIntegrator::endBlock (C++ function)  OpenMM::CustomIntegrator::getComputationStep (C++ function)  OpenMM::CustomIntegrator::getGlobalVariable (C++ function)  OpenMM::CustomIntegrator::getGlobalVariableByName (C++ function)  OpenMM::CustomIntegrator::getGlobalVariableName (C++ function)  OpenMM::CustomIntegrator::getKineticEnergyExpression (C++ function)  OpenMM::CustomIntegrator::getNumComputations (C++ function)  OpenMM::CustomIntegrator::getNumGlobalVariables (C++ function)  OpenMM::CustomIntegrator::getNumPerDofVariables (C++ function)  OpenMM::CustomIntegrator::getPerDofVariable (C++ function)  OpenMM::CustomIntegrator::getPerDofVariableByName (C++ function)  OpenMM::CustomIntegrator::getPerDofVariableName (C++ function)  OpenMM::CustomIntegrator::getRandomNumberSeed (C++ function) | OpenMM::CustomIntegrator::setGlobalVariable (C++ function)  OpenMM::CustomIntegrator::setGlobalVariableByName (C++ function)  OpenMM::CustomIntegrator::setKineticEnergyExpression (C++ function)  OpenMM::CustomIntegrator::setPerDofVariable (C++ function)  OpenMM::CustomIntegrator::setPerDofVariableByName (C++ function)  OpenMM::CustomIntegrator::setRandomNumberSeed (C++ function)  OpenMM::CustomIntegrator::step (C++ function)  OpenMM::CustomManyParticleForce (C++ class)  OpenMM::CustomManyParticleForce::addExclusion (C++ function)  OpenMM::CustomManyParticleForce::addGlobalParameter (C++ function)  OpenMM::CustomManyParticleForce::addParticle (C++ function)  OpenMM::CustomManyParticleForce::addPerParticleParameter (C++ function)  OpenMM::CustomManyParticleForce::addTabulatedFunction (C++ function)  OpenMM::CustomManyParticleForce::createExclusionsFromBonds (C++ function)  OpenMM::CustomManyParticleForce::CustomManyParticleForce (C++ function)  OpenMM::CustomManyParticleForce::getCutoffDistance (C++ function)  OpenMM::CustomManyParticleForce::getEnergyFunction (C++ function)  OpenMM::CustomManyParticleForce::getExclusionParticles (C++ function)  OpenMM::CustomManyParticleForce::getGlobalParameterDefaultValue (C++ function)  OpenMM::CustomManyParticleForce::getGlobalParameterName (C++ function)  OpenMM::CustomManyParticleForce::getNonbondedMethod (C++ function)  OpenMM::CustomManyParticleForce::getNumExclusions (C++ function)  OpenMM::CustomManyParticleForce::getNumGlobalParameters (C++ function)  OpenMM::CustomManyParticleForce::getNumParticles (C++ function)  OpenMM::CustomManyParticleForce::getNumParticlesPerSet (C++ function)  OpenMM::CustomManyParticleForce::getNumPerParticleParameters (C++ function)  OpenMM::CustomManyParticleForce::getNumTabulatedFunctions (C++ function)  OpenMM::CustomManyParticleForce::getParticleParameters (C++ function)  OpenMM::CustomManyParticleForce::getPermutationMode (C++ function)  OpenMM::CustomManyParticleForce::getPerParticleParameterName (C++ function)  OpenMM::CustomManyParticleForce::getTabulatedFunction (C++ function), [1]  OpenMM::CustomManyParticleForce::getTabulatedFunctionName (C++ function)  OpenMM::CustomManyParticleForce::getTypeFilter (C++ function)  OpenMM::CustomManyParticleForce::setCutoffDistance (C++ function)  OpenMM::CustomManyParticleForce::setEnergyFunction (C++ function)  OpenMM::CustomManyParticleForce::setExclusionParticles (C++ function)  OpenMM::CustomManyParticleForce::setGlobalParameterDefaultValue (C++ function)  OpenMM::CustomManyParticleForce::setGlobalParameterName (C++ function)  OpenMM::CustomManyParticleForce::setNonbondedMethod (C++ function)  OpenMM::CustomManyParticleForce::setParticleParameters (C++ function)  OpenMM::CustomManyParticleForce::setPermutationMode (C++ function)  OpenMM::CustomManyParticleForce::setPerParticleParameterName (C++ function)  OpenMM::CustomManyParticleForce::setTypeFilter (C++ function)  OpenMM::CustomManyParticleForce::updateParametersInContext (C++ function)  OpenMM::CustomManyParticleForce::usesPeriodicBoundaryConditions (C++ function)  OpenMM::CustomManyParticleForce::~CustomManyParticleForce (C++ function)  OpenMM::CustomNonbondedForce (C++ class)  OpenMM::CustomNonbondedForce::addExclusion (C++ function)  OpenMM::CustomNonbondedForce::addFunction (C++ function)  OpenMM::CustomNonbondedForce::addGlobalParameter (C++ function)  OpenMM::CustomNonbondedForce::addInteractionGroup (C++ function)  OpenMM::CustomNonbondedForce::addParticle (C++ function)  OpenMM::CustomNonbondedForce::addPerParticleParameter (C++ function)  OpenMM::CustomNonbondedForce::addTabulatedFunction (C++ function)  OpenMM::CustomNonbondedForce::createExclusionsFromBonds (C++ function)  OpenMM::CustomNonbondedForce::CustomNonbondedForce (C++ function), [1]  OpenMM::CustomNonbondedForce::getCutoffDistance (C++ function)  OpenMM::CustomNonbondedForce::getEnergyFunction (C++ function)  OpenMM::CustomNonbondedForce::getExclusionParticles (C++ function)  OpenMM::CustomNonbondedForce::getFunctionParameters (C++ function)  OpenMM::CustomNonbondedForce::getGlobalParameterDefaultValue (C++ function)  OpenMM::CustomNonbondedForce::getGlobalParameterName (C++ function)  OpenMM::CustomNonbondedForce::getInteractionGroupParameters (C++ function)  OpenMM::CustomNonbondedForce::getNonbondedMethod (C++ function)  OpenMM::CustomNonbondedForce::getNumExclusions (C++ function)  OpenMM::CustomNonbondedForce::getNumFunctions (C++ function)  OpenMM::CustomNonbondedForce::getNumGlobalParameters (C++ function)  OpenMM::CustomNonbondedForce::getNumInteractionGroups (C++ function)  OpenMM::CustomNonbondedForce::getNumParticles (C++ function)  OpenMM::CustomNonbondedForce::getNumPerParticleParameters (C++ function)  OpenMM::CustomNonbondedForce::getNumTabulatedFunctions (C++ function)  OpenMM::CustomNonbondedForce::getParticleParameters (C++ function)  OpenMM::CustomNonbondedForce::getPerParticleParameterName (C++ function)  OpenMM::CustomNonbondedForce::getSwitchingDistance (C++ function)  OpenMM::CustomNonbondedForce::getTabulatedFunction (C++ function), [1]  OpenMM::CustomNonbondedForce::getTabulatedFunctionName (C++ function)  OpenMM::CustomNonbondedForce::getUseLongRangeCorrection (C++ function)  OpenMM::CustomNonbondedForce::getUseSwitchingFunction (C++ function)  OpenMM::CustomNonbondedForce::setCutoffDistance (C++ function)  OpenMM::CustomNonbondedForce::setEnergyFunction (C++ function)  OpenMM::CustomNonbondedForce::setExclusionParticles (C++ function)  OpenMM::CustomNonbondedForce::setFunctionParameters (C++ function)  OpenMM::CustomNonbondedForce::setGlobalParameterDefaultValue (C++ function)  OpenMM::CustomNonbondedForce::setGlobalParameterName (C++ function)  OpenMM::CustomNonbondedForce::setInteractionGroupParameters (C++ function)  OpenMM::CustomNonbondedForce::setNonbondedMethod (C++ function)  OpenMM::CustomNonbondedForce::setParticleParameters (C++ function)  OpenMM::CustomNonbondedForce::setPerParticleParameterName (C++ function)  OpenMM::CustomNonbondedForce::setSwitchingDistance (C++ function)  OpenMM::CustomNonbondedForce::setUseLongRangeCorrection (C++ function)  OpenMM::CustomNonbondedForce::setUseSwitchingFunction (C++ function)  OpenMM::CustomNonbondedForce::updateParametersInContext (C++ function)  OpenMM::CustomNonbondedForce::usesPeriodicBoundaryConditions (C++ function)  OpenMM::CustomNonbondedForce::~CustomNonbondedForce (C++ function)  OpenMM::CustomTorsionForce (C++ class)  OpenMM::CustomTorsionForce::addGlobalParameter (C++ function)  OpenMM::CustomTorsionForce::addPerTorsionParameter (C++ function)  OpenMM::CustomTorsionForce::addTorsion (C++ function)  OpenMM::CustomTorsionForce::CustomTorsionForce (C++ function)  OpenMM::CustomTorsionForce::getEnergyFunction (C++ function)  OpenMM::CustomTorsionForce::getGlobalParameterDefaultValue (C++ function)  OpenMM::CustomTorsionForce::getGlobalParameterName (C++ function)  OpenMM::CustomTorsionForce::getNumGlobalParameters (C++ function)  OpenMM::CustomTorsionForce::getNumPerTorsionParameters (C++ function)  OpenMM::CustomTorsionForce::getNumTorsions (C++ function)  OpenMM::CustomTorsionForce::getPerTorsionParameterName (C++ function)  OpenMM::CustomTorsionForce::getTorsionParameters (C++ function)  OpenMM::CustomTorsionForce::setEnergyFunction (C++ function)  OpenMM::CustomTorsionForce::setGlobalParameterDefaultValue (C++ function)  OpenMM::CustomTorsionForce::setGlobalParameterName (C++ function)  OpenMM::CustomTorsionForce::setPerTorsionParameterName (C++ function)  OpenMM::CustomTorsionForce::setTorsionParameters (C++ function)  OpenMM::CustomTorsionForce::updateParametersInContext (C++ function)  OpenMM::CustomTorsionForce::usesPeriodicBoundaryConditions (C++ function)  OpenMM::Discrete1DFunction (C++ class)  OpenMM::Discrete1DFunction::Copy (C++ function)  OpenMM::Discrete1DFunction::Discrete1DFunction (C++ function)  OpenMM::Discrete1DFunction::getFunctionParameters (C++ function)  OpenMM::Discrete1DFunction::setFunctionParameters (C++ function)  OpenMM::Discrete2DFunction (C++ class)  OpenMM::Discrete2DFunction::Copy (C++ function)  OpenMM::Discrete2DFunction::Discrete2DFunction (C++ function)  OpenMM::Discrete2DFunction::getFunctionParameters (C++ function)  OpenMM::Discrete2DFunction::setFunctionParameters (C++ function)  OpenMM::Discrete3DFunction (C++ class)  OpenMM::Discrete3DFunction::Copy (C++ function)  OpenMM::Discrete3DFunction::Discrete3DFunction (C++ function)  OpenMM::Discrete3DFunction::getFunctionParameters (C++ function)  OpenMM::Discrete3DFunction::setFunctionParameters (C++ function)  OpenMM::DrudeForce (C++ class)  OpenMM::DrudeForce::addParticle (C++ function)  OpenMM::DrudeForce::addScreenedPair (C++ function)  OpenMM::DrudeForce::DrudeForce (C++ function)  OpenMM::DrudeForce::getNumParticles (C++ function)  OpenMM::DrudeForce::getNumScreenedPairs (C++ function)  OpenMM::DrudeForce::getParticleParameters (C++ function)  OpenMM::DrudeForce::getScreenedPairParameters (C++ function)  OpenMM::DrudeForce::setParticleParameters (C++ function)  OpenMM::DrudeForce::setScreenedPairParameters (C++ function)  OpenMM::DrudeForce::updateParametersInContext (C++ function)  OpenMM::DrudeForce::usesPeriodicBoundaryConditions (C++ function)  OpenMM::DrudeLangevinIntegrator (C++ class)  OpenMM::DrudeLangevinIntegrator::DrudeLangevinIntegrator (C++ function)  OpenMM::DrudeLangevinIntegrator::getDrudeFriction (C++ function)  OpenMM::DrudeLangevinIntegrator::getDrudeTemperature (C++ function)  OpenMM::DrudeLangevinIntegrator::getFriction (C++ function)  OpenMM::DrudeLangevinIntegrator::getMaxDrudeDistance (C++ function)  OpenMM::DrudeLangevinIntegrator::getRandomNumberSeed (C++ function)  OpenMM::DrudeLangevinIntegrator::getTemperature (C++ function)  OpenMM::DrudeLangevinIntegrator::setDrudeFriction (C++ function)  OpenMM::DrudeLangevinIntegrator::setDrudeTemperature (C++ function)  OpenMM::DrudeLangevinIntegrator::setFriction (C++ function)  OpenMM::DrudeLangevinIntegrator::setMaxDrudeDistance (C++ function)  OpenMM::DrudeLangevinIntegrator::setRandomNumberSeed (C++ function)  OpenMM::DrudeLangevinIntegrator::setTemperature (C++ function)  OpenMM::DrudeLangevinIntegrator::step (C++ function)  OpenMM::DrudeSCFIntegrator (C++ class)  OpenMM::DrudeSCFIntegrator::DrudeSCFIntegrator (C++ function)  OpenMM::DrudeSCFIntegrator::getMinimizationErrorTolerance (C++ function)  OpenMM::DrudeSCFIntegrator::setMinimizationErrorTolerance (C++ function)  OpenMM::DrudeSCFIntegrator::step (C++ function)  OpenMM::Force (C++ class)  OpenMM::Force::Force (C++ function)  OpenMM::Force::getForceGroup (C++ function)  OpenMM::Force::setForceGroup (C++ function)  OpenMM::Force::usesPeriodicBoundaryConditions (C++ function)  OpenMM::Force::~Force (C++ function)  OpenMM::GBSAOBCForce (C++ class)  OpenMM::GBSAOBCForce::addParticle (C++ function)  OpenMM::GBSAOBCForce::GBSAOBCForce (C++ function)  OpenMM::GBSAOBCForce::getCutoffDistance (C++ function)  OpenMM::GBSAOBCForce::getNonbondedMethod (C++ function)  OpenMM::GBSAOBCForce::getNumParticles (C++ function)  OpenMM::GBSAOBCForce::getParticleParameters (C++ function)  OpenMM::GBSAOBCForce::getSoluteDielectric (C++ function)  OpenMM::GBSAOBCForce::getSolventDielectric (C++ function)  OpenMM::GBSAOBCForce::getSurfaceAreaEnergy (C++ function)  OpenMM::GBSAOBCForce::setCutoffDistance (C++ function)  OpenMM::GBSAOBCForce::setNonbondedMethod (C++ function)  OpenMM::GBSAOBCForce::setParticleParameters (C++ function)  OpenMM::GBSAOBCForce::setSoluteDielectric (C++ function)  OpenMM::GBSAOBCForce::setSolventDielectric (C++ function)  OpenMM::GBSAOBCForce::setSurfaceAreaEnergy (C++ function)  OpenMM::GBSAOBCForce::updateParametersInContext (C++ function)  OpenMM::GBSAOBCForce::usesPeriodicBoundaryConditions (C++ function)  OpenMM::HarmonicAngleForce (C++ class)  OpenMM::HarmonicAngleForce::addAngle (C++ function)  OpenMM::HarmonicAngleForce::getAngleParameters (C++ function)  OpenMM::HarmonicAngleForce::getNumAngles (C++ function)  OpenMM::HarmonicAngleForce::HarmonicAngleForce (C++ function)  OpenMM::HarmonicAngleForce::setAngleParameters (C++ function)  OpenMM::HarmonicAngleForce::updateParametersInContext (C++ function)  OpenMM::HarmonicAngleForce::usesPeriodicBoundaryConditions (C++ function)  OpenMM::HarmonicBondForce (C++ class)  OpenMM::HarmonicBondForce::addBond (C++ function)  OpenMM::HarmonicBondForce::getBondParameters (C++ function)  OpenMM::HarmonicBondForce::getNumBonds (C++ function)  OpenMM::HarmonicBondForce::HarmonicBondForce (C++ function)  OpenMM::HarmonicBondForce::setBondParameters (C++ function)  OpenMM::HarmonicBondForce::updateParametersInContext (C++ function)  OpenMM::HarmonicBondForce::usesPeriodicBoundaryConditions (C++ function)  OpenMM::Integrator (C++ class)  OpenMM::Integrator::getConstraintTolerance (C++ function)  OpenMM::Integrator::getStepSize (C++ function)  OpenMM::Integrator::Integrator (C++ function)  OpenMM::Integrator::setConstraintTolerance (C++ function)  OpenMM::Integrator::setStepSize (C++ function)  OpenMM::Integrator::step (C++ function)  OpenMM::Integrator::~Integrator (C++ function)  OpenMM::LangevinIntegrator (C++ class)  OpenMM::LangevinIntegrator::getFriction (C++ function)  OpenMM::LangevinIntegrator::getRandomNumberSeed (C++ function)  OpenMM::LangevinIntegrator::getTemperature (C++ function)  OpenMM::LangevinIntegrator::LangevinIntegrator (C++ function)  OpenMM::LangevinIntegrator::setFriction (C++ function)  OpenMM::LangevinIntegrator::setRandomNumberSeed (C++ function)  OpenMM::LangevinIntegrator::setTemperature (C++ function)  OpenMM::LangevinIntegrator::step (C++ function)  OpenMM::LocalCoordinatesSite (C++ class)  OpenMM::LocalCoordinatesSite::getLocalPosition (C++ function)  OpenMM::LocalCoordinatesSite::getOriginWeights (C++ function)  OpenMM::LocalCoordinatesSite::getXWeights (C++ function)  OpenMM::LocalCoordinatesSite::getYWeights (C++ function)  OpenMM::LocalCoordinatesSite::LocalCoordinatesSite (C++ function)  OpenMM::LocalEnergyMinimizer (C++ class)  OpenMM::LocalEnergyMinimizer::minimize (C++ function)  OpenMM::MonteCarloAnisotropicBarostat (C++ class)  OpenMM::MonteCarloAnisotropicBarostat::getDefaultPressure (C++ function)  OpenMM::MonteCarloAnisotropicBarostat::getFrequency (C++ function)  OpenMM::MonteCarloAnisotropicBarostat::getRandomNumberSeed (C++ function)  OpenMM::MonteCarloAnisotropicBarostat::getScaleX (C++ function)  OpenMM::MonteCarloAnisotropicBarostat::getScaleY (C++ function)  OpenMM::MonteCarloAnisotropicBarostat::getScaleZ (C++ function)  OpenMM::MonteCarloAnisotropicBarostat::getTemperature (C++ function)  OpenMM::MonteCarloAnisotropicBarostat::MonteCarloAnisotropicBarostat (C++ function)  OpenMM::MonteCarloAnisotropicBarostat::PressureX (C++ function)  OpenMM::MonteCarloAnisotropicBarostat::PressureY (C++ function)  OpenMM::MonteCarloAnisotropicBarostat::PressureZ (C++ function)  OpenMM::MonteCarloAnisotropicBarostat::setDefaultPressure (C++ function)  OpenMM::MonteCarloAnisotropicBarostat::setFrequency (C++ function)  OpenMM::MonteCarloAnisotropicBarostat::setRandomNumberSeed (C++ function)  OpenMM::MonteCarloAnisotropicBarostat::setTemperature (C++ function)  OpenMM::MonteCarloAnisotropicBarostat::usesPeriodicBoundaryConditions (C++ function)  OpenMM::MonteCarloBarostat (C++ class)  OpenMM::MonteCarloBarostat::getDefaultPressure (C++ function)  OpenMM::MonteCarloBarostat::getFrequency (C++ function)  OpenMM::MonteCarloBarostat::getRandomNumberSeed (C++ function)  OpenMM::MonteCarloBarostat::getTemperature (C++ function)  OpenMM::MonteCarloBarostat::MonteCarloBarostat (C++ function)  OpenMM::MonteCarloBarostat::Pressure (C++ function)  OpenMM::MonteCarloBarostat::setDefaultPressure (C++ function)  OpenMM::MonteCarloBarostat::setFrequency (C++ function)  OpenMM::MonteCarloBarostat::setRandomNumberSeed (C++ function)  OpenMM::MonteCarloBarostat::setTemperature (C++ function)  OpenMM::MonteCarloBarostat::usesPeriodicBoundaryConditions (C++ function)  OpenMM::MonteCarloMembraneBarostat (C++ class)  OpenMM::MonteCarloMembraneBarostat::getDefaultPressure (C++ function)  OpenMM::MonteCarloMembraneBarostat::getDefaultSurfaceTension (C++ function)  OpenMM::MonteCarloMembraneBarostat::getFrequency (C++ function)  OpenMM::MonteCarloMembraneBarostat::getRandomNumberSeed (C++ function)  OpenMM::MonteCarloMembraneBarostat::getTemperature (C++ function)  OpenMM::MonteCarloMembraneBarostat::getXYMode (C++ function)  OpenMM::MonteCarloMembraneBarostat::getZMode (C++ function)  OpenMM::MonteCarloMembraneBarostat::MonteCarloMembraneBarostat (C++ function)  OpenMM::MonteCarloMembraneBarostat::Pressure (C++ function)  OpenMM::MonteCarloMembraneBarostat::setDefaultPressure (C++ function)  OpenMM::MonteCarloMembraneBarostat::setDefaultSurfaceTension (C++ function)  OpenMM::MonteCarloMembraneBarostat::setFrequency (C++ function)  OpenMM::MonteCarloMembraneBarostat::setRandomNumberSeed (C++ function)  OpenMM::MonteCarloMembraneBarostat::setTemperature (C++ function)  OpenMM::MonteCarloMembraneBarostat::setXYMode (C++ function)  OpenMM::MonteCarloMembraneBarostat::setZMode (C++ function)  OpenMM::MonteCarloMembraneBarostat::SurfaceTension (C++ function)  OpenMM::MonteCarloMembraneBarostat::usesPeriodicBoundaryConditions (C++ function)  OpenMM::NonbondedForce (C++ class)  OpenMM::NonbondedForce::addException (C++ function)  OpenMM::NonbondedForce::addParticle (C++ function)  OpenMM::NonbondedForce::createExceptionsFromBonds (C++ function)  OpenMM::NonbondedForce::getCutoffDistance (C++ function)  OpenMM::NonbondedForce::getEwaldErrorTolerance (C++ function)  OpenMM::NonbondedForce::getExceptionParameters (C++ function)  OpenMM::NonbondedForce::getNonbondedMethod (C++ function)  OpenMM::NonbondedForce::getNumExceptions (C++ function)  OpenMM::NonbondedForce::getNumParticles (C++ function)  OpenMM::NonbondedForce::getParticleParameters (C++ function)  OpenMM::NonbondedForce::getPMEParameters (C++ function)  OpenMM::NonbondedForce::getPMEParametersInContext (C++ function)  OpenMM::NonbondedForce::getReactionFieldDielectric (C++ function)  OpenMM::NonbondedForce::getReciprocalSpaceForceGroup (C++ function)  OpenMM::NonbondedForce::getSwitchingDistance (C++ function)  OpenMM::NonbondedForce::getUseDispersionCorrection (C++ function)  OpenMM::NonbondedForce::getUseSwitchingFunction (C++ function)  OpenMM::NonbondedForce::NonbondedForce (C++ function)  OpenMM::NonbondedForce::setCutoffDistance (C++ function)  OpenMM::NonbondedForce::setEwaldErrorTolerance (C++ function)  OpenMM::NonbondedForce::setExceptionParameters (C++ function)  OpenMM::NonbondedForce::setNonbondedMethod (C++ function)  OpenMM::NonbondedForce::setParticleParameters (C++ function)  OpenMM::NonbondedForce::setPMEParameters (C++ function)  OpenMM::NonbondedForce::setReactionFieldDielectric (C++ function)  OpenMM::NonbondedForce::setReciprocalSpaceForceGroup (C++ function)  OpenMM::NonbondedForce::setSwitchingDistance (C++ function)  OpenMM::NonbondedForce::setUseDispersionCorrection (C++ function)  OpenMM::NonbondedForce::setUseSwitchingFunction (C++ function)  OpenMM::NonbondedForce::updateParametersInContext (C++ function)  OpenMM::NonbondedForce::usesPeriodicBoundaryConditions (C++ function)  OpenMM::OpenMMException (C++ class)  OpenMM::OpenMMException::OpenMMException (C++ function)  OpenMM::OpenMMException::what (C++ function)  OpenMM::OpenMMException::~OpenMMException (C++ function)  OpenMM::OutOfPlaneSite (C++ class)  OpenMM::OutOfPlaneSite::getWeight12 (C++ function)  OpenMM::OutOfPlaneSite::getWeight13 (C++ function)  OpenMM::OutOfPlaneSite::getWeightCross (C++ function)  OpenMM::OutOfPlaneSite::OutOfPlaneSite (C++ function)  OpenMM::PeriodicTorsionForce (C++ class)  OpenMM::PeriodicTorsionForce::addTorsion (C++ function)  OpenMM::PeriodicTorsionForce::getNumTorsions (C++ function)  OpenMM::PeriodicTorsionForce::getTorsionParameters (C++ function)  OpenMM::PeriodicTorsionForce::PeriodicTorsionForce (C++ function)  OpenMM::PeriodicTorsionForce::setTorsionParameters (C++ function)  OpenMM::PeriodicTorsionForce::updateParametersInContext (C++ function)  OpenMM::PeriodicTorsionForce::usesPeriodicBoundaryConditions (C++ function)  OpenMM::Platform (C++ class)  OpenMM::Platform::contextCreated (C++ function)  OpenMM::Platform::contextDestroyed (C++ function)  OpenMM::Platform::createKernel (C++ function)  OpenMM::Platform::findPlatform (C++ function)  OpenMM::Platform::getDefaultPluginsDirectory (C++ function)  OpenMM::Platform::getName (C++ function)  OpenMM::Platform::getNumPlatforms (C++ function)  OpenMM::Platform::getOpenMMVersion (C++ function)  OpenMM::Platform::getPlatform (C++ function)  OpenMM::Platform::getPlatformByName (C++ function)  OpenMM::Platform::getPluginLoadFailures (C++ function)  OpenMM::Platform::getPropertyDefaultValue (C++ function)  OpenMM::Platform::getPropertyNames (C++ function)  OpenMM::Platform::getPropertyValue (C++ function)  OpenMM::Platform::getSpeed (C++ function)  OpenMM::Platform::loadPluginLibrary (C++ function)  OpenMM::Platform::loadPluginsFromDirectory (C++ function)  OpenMM::Platform::registerKernelFactory (C++ function)  OpenMM::Platform::registerPlatform (C++ function)  OpenMM::Platform::setPropertyDefaultValue (C++ function)  OpenMM::Platform::setPropertyValue (C++ function)  OpenMM::Platform::supportsDoublePrecision (C++ function)  OpenMM::Platform::supportsKernels (C++ function)  OpenMM::Platform::~Platform (C++ function)  OpenMM::RBTorsionForce (C++ class)  OpenMM::RBTorsionForce::addTorsion (C++ function)  OpenMM::RBTorsionForce::getNumTorsions (C++ function)  OpenMM::RBTorsionForce::getTorsionParameters (C++ function)  OpenMM::RBTorsionForce::RBTorsionForce (C++ function)  OpenMM::RBTorsionForce::setTorsionParameters (C++ function)  OpenMM::RBTorsionForce::updateParametersInContext (C++ function)  OpenMM::RBTorsionForce::usesPeriodicBoundaryConditions (C++ function)  OpenMM::RPMDIntegrator (C++ class)  OpenMM::RPMDIntegrator::getApplyThermostat (C++ function)  OpenMM::RPMDIntegrator::getContractions (C++ function)  OpenMM::RPMDIntegrator::getFriction (C++ function)  OpenMM::RPMDIntegrator::getNumCopies (C++ function)  OpenMM::RPMDIntegrator::getRandomNumberSeed (C++ function)  OpenMM::RPMDIntegrator::getState (C++ function)  OpenMM::RPMDIntegrator::getTemperature (C++ function)  OpenMM::RPMDIntegrator::getTotalEnergy (C++ function)  OpenMM::RPMDIntegrator::RPMDIntegrator (C++ function), [1]  OpenMM::RPMDIntegrator::setApplyThermostat (C++ function)  OpenMM::RPMDIntegrator::setFriction (C++ function)  OpenMM::RPMDIntegrator::setPositions (C++ function)  OpenMM::RPMDIntegrator::setRandomNumberSeed (C++ function)  OpenMM::RPMDIntegrator::setTemperature (C++ function)  OpenMM::RPMDIntegrator::setVelocities (C++ function)  OpenMM::RPMDIntegrator::step (C++ function)  OpenMM::RPMDMonteCarloBarostat (C++ class)  OpenMM::RPMDMonteCarloBarostat::getDefaultPressure (C++ function)  OpenMM::RPMDMonteCarloBarostat::getFrequency (C++ function)  OpenMM::RPMDMonteCarloBarostat::getRandomNumberSeed (C++ function)  OpenMM::RPMDMonteCarloBarostat::Pressure (C++ function)  OpenMM::RPMDMonteCarloBarostat::RPMDMonteCarloBarostat (C++ function)  OpenMM::RPMDMonteCarloBarostat::setDefaultPressure (C++ function)  OpenMM::RPMDMonteCarloBarostat::setFrequency (C++ function)  OpenMM::RPMDMonteCarloBarostat::setRandomNumberSeed (C++ function)  OpenMM::RPMDMonteCarloBarostat::usesPeriodicBoundaryConditions (C++ function)  OpenMM::SerializationNode (C++ class)  OpenMM::SerializationNode::createChildNode (C++ function), [1]  OpenMM::SerializationNode::decodeObject (C++ function)  OpenMM::SerializationNode::getBoolProperty (C++ function), [1]  OpenMM::SerializationNode::getChildNode (C++ function), [1]  OpenMM::SerializationNode::getChildren (C++ function), [1]  OpenMM::SerializationNode::getDoubleProperty (C++ function), [1]  OpenMM::SerializationNode::getIntProperty (C++ function), [1]  OpenMM::SerializationNode::getName (C++ function)  OpenMM::SerializationNode::getProperties (C++ function)  OpenMM::SerializationNode::getStringProperty (C++ function), [1]  OpenMM::SerializationNode::hasProperty (C++ function)  OpenMM::SerializationNode::setBoolProperty (C++ function)  OpenMM::SerializationNode::setDoubleProperty (C++ function)  OpenMM::SerializationNode::setIntProperty (C++ function)  OpenMM::SerializationNode::setName (C++ function)  OpenMM::SerializationNode::setStringProperty (C++ function)  OpenMM::SerializationProxy (C++ class)  OpenMM::SerializationProxy::deserialize (C++ function)  OpenMM::SerializationProxy::getProxy (C++ function), [1]  OpenMM::SerializationProxy::getTypeName (C++ function)  OpenMM::SerializationProxy::registerProxy (C++ function)  OpenMM::SerializationProxy::SerializationProxy (C++ function)  OpenMM::SerializationProxy::serialize (C++ function)  OpenMM::SerializationProxy::~SerializationProxy (C++ function)  OpenMM::State (C++ class)  OpenMM::State::getDataTypes (C++ function)  OpenMM::State::getForces (C++ function)  OpenMM::State::getKineticEnergy (C++ function)  OpenMM::State::getParameters (C++ function)  OpenMM::State::getPeriodicBoxVectors (C++ function)  OpenMM::State::getPeriodicBoxVolume (C++ function)  OpenMM::State::getPositions (C++ function)  OpenMM::State::getPotentialEnergy (C++ function)  OpenMM::State::getState (C++ function)  OpenMM::State::getTime (C++ function)  OpenMM::State::getVelocities (C++ function)  OpenMM::State::setEnergy (C++ function)  OpenMM::State::setForces (C++ function)  OpenMM::State::setParameters (C++ function)  OpenMM::State::setPeriodicBoxVectors (C++ function)  OpenMM::State::setPositions (C++ function)  OpenMM::State::setVelocities (C++ function)  OpenMM::State::State (C++ function)  OpenMM::State::StateBuilder (C++ function)  OpenMM::System (C++ class)  OpenMM::System::addConstraint (C++ function)  OpenMM::System::addForce (C++ function)  OpenMM::System::addParticle (C++ function)  OpenMM::System::getConstraintParameters (C++ function)  OpenMM::System::getDefaultPeriodicBoxVectors (C++ function)  OpenMM::System::getForce (C++ function), [1]  OpenMM::System::getNumConstraints (C++ function)  OpenMM::System::getNumForces (C++ function)  OpenMM::System::getNumParticles (C++ function)  OpenMM::System::getParticleMass (C++ function)  OpenMM::System::getVirtualSite (C++ function)  OpenMM::System::isVirtualSite (C++ function)  OpenMM::System::removeConstraint (C++ function)  OpenMM::System::removeForce (C++ function)  OpenMM::System::setConstraintParameters (C++ function)  OpenMM::System::setDefaultPeriodicBoxVectors (C++ function)  OpenMM::System::setParticleMass (C++ function)  OpenMM::System::setVirtualSite (C++ function)  OpenMM::System::System (C++ function)  OpenMM::System::usesPeriodicBoundaryConditions (C++ function)  OpenMM::System::~System (C++ function)  OpenMM::TabulatedFunction (C++ class)  OpenMM::TabulatedFunction::Copy (C++ function)  OpenMM::TabulatedFunction::~TabulatedFunction (C++ function)  OpenMM::ThreeParticleAverageSite (C++ class)  OpenMM::ThreeParticleAverageSite::getWeight (C++ function)  OpenMM::ThreeParticleAverageSite::ThreeParticleAverageSite (C++ function)  OpenMM::TwoParticleAverageSite (C++ class)  OpenMM::TwoParticleAverageSite::getWeight (C++ function)  OpenMM::TwoParticleAverageSite::TwoParticleAverageSite (C++ function)  OpenMM::VariableLangevinIntegrator (C++ class)  OpenMM::VariableLangevinIntegrator::getErrorTolerance (C++ function)  OpenMM::VariableLangevinIntegrator::getFriction (C++ function)  OpenMM::VariableLangevinIntegrator::getRandomNumberSeed (C++ function)  OpenMM::VariableLangevinIntegrator::getTemperature (C++ function)  OpenMM::VariableLangevinIntegrator::setErrorTolerance (C++ function)  OpenMM::VariableLangevinIntegrator::setFriction (C++ function)  OpenMM::VariableLangevinIntegrator::setRandomNumberSeed (C++ function)  OpenMM::VariableLangevinIntegrator::setTemperature (C++ function)  OpenMM::VariableLangevinIntegrator::step (C++ function)  OpenMM::VariableLangevinIntegrator::stepTo (C++ function)  OpenMM::VariableLangevinIntegrator::VariableLangevinIntegrator (C++ function)  OpenMM::VariableVerletIntegrator (C++ class)  OpenMM::VariableVerletIntegrator::getErrorTolerance (C++ function)  OpenMM::VariableVerletIntegrator::setErrorTolerance (C++ function)  OpenMM::VariableVerletIntegrator::step (C++ function)  OpenMM::VariableVerletIntegrator::stepTo (C++ function)  OpenMM::VariableVerletIntegrator::VariableVerletIntegrator (C++ function)  OpenMM::Vec3 (C++ class)  OpenMM::Vec3::cross (C++ function)  OpenMM::Vec3::dot (C++ function)  OpenMM::Vec3::operator!= (C++ function)  OpenMM::Vec3::operator\* (C++ function)  OpenMM::Vec3::operator\*= (C++ function)  OpenMM::Vec3::operator+ (C++ function), [1]  OpenMM::Vec3::operator+= (C++ function)  OpenMM::Vec3::operator- (C++ function), [1]  OpenMM::Vec3::operator-= (C++ function)  OpenMM::Vec3::operator/ (C++ function)  OpenMM::Vec3::operator/= (C++ function)  OpenMM::Vec3::operator== (C++ function)  OpenMM::Vec3::operator[] (C++ function), [1]  OpenMM::Vec3::Vec3 (C++ function), [1]  OpenMM::VerletIntegrator (C++ class)  OpenMM::VerletIntegrator::step (C++ function)  OpenMM::VerletIntegrator::VerletIntegrator (C++ function)  OpenMM::VirtualSite (C++ class)  OpenMM::VirtualSite::getNumParticles (C++ function)  OpenMM::VirtualSite::getParticle (C++ function)  OpenMM::VirtualSite::~VirtualSite (C++ function)  OpenMM::XmlSerializer (C++ class)  OpenMM::XmlSerializer::clone (C++ function)  OpenMM::XmlSerializer::deserialize (C++ function)  OpenMM::XmlSerializer::serialize (C++ function) |

High performance molecular simulation on GPUs


### Navigation

- Core Objects
- Forces
- Integrators
- Extras

©2015, Stanford University and the Authors.
|
Powered by Sphinx 1.3.1
& Alabaster 0.7.7
